# The Value of Ultrasonography in determining Pupillary Light Reflex in Patients with Traumatic Ocular Injuries; a Letter to Editor

**Published:** 2019-11-10

**Authors:** Hamid Mirjalili, Ali Raee Ezzabadi, Yeganeh Yazdiyousefi, Mohammad Ali Jafari Nadoshan

**Affiliations:** 1 Emergency Department, Shahid Sadoughi University of Medical Science, Yazd, Iran.

**Keywords:** Ultrasonography, Reflex, Pupillary, Hyphema, Hematoma, Eye Injuries

Pupillary light reflex (PLR) measurement is one of the frequent physical examinations used by emergency physicians for assessment of brain stem function and monitoring of intracranial pressure (1, 2). PLR could be abnormal due to various causes such as optic nerve damage, oculomotor nerve damage, brain stem lesion, and using central nervous system depressant drugs, such as barbiturates.

Direct PLR measurement is not always possible in patients with ocular trauma suffering from hematoma, hyphema, and etc. One helpful tool for measuring PLR in this situation is ultrasonography (3, 4). Using a coronal ultrasonographic view of the iris upon contralateral stimulation with a penlight, Ashot et al. emphasized that ultrasonography could be considered as a practical, fast and recordable method for evaluating PLR (5). 

Using consecutive sampling, the authors of this letter examined all head trauma patients suffering from unilateral ocular trauma, who visited the emergency departments of Shahid Sadooghi and Shahid Rahnemoun hospitals, Yazd, Iran, from 2017 to 2018 (ethics code: IR.SSU.MEDICINE.REC.1396.265). Patients with bilateral ocular trauma, penetrating trauma, history of any chronic disease (glaucoma, cataract, retina, etc.), or previous eye surgeries were excluded.

Using EZONO 3000 ultrasound system and a linear probe with a 10 MHz frequency in coronal sections, PLR was measured in both intact and injured eyes. The probe is fanned until the iris and pupil are visualized and a bright light is flashed (distance 1 cm).

The pupil sizes for both intact and injured eyes were measured and registered through physical examination and ultrasonography before and after flashing light to the eye, separately (PLR in injured eye was measured using indirect light and light was flashed into the intact eye). Then, the initial pupil size and PLR were compared between physical and ultrasonography examinations (The study was performed on both conscious and unconscious patients). 

Finally, 74 cases with the mean age of 20.30 ± 44.9 years were studied (87.8% male). In physical examination, patients’ pupillary response was unclear in 17 cases, light response occurred in 55 cases, and two patients didn’t exhibit pupillary response. However, examining the patients’ pupils through ultrasonography, the PLR in one case was unclear and 62 individuals exhibited pupillary response, while 11 did not. 

There was a significant difference between physical examination and ultrasonography findings regarding the pupil size of injured eyes (2.82 ± 1.63 vs 3.58 ± 0.95, respectively; p = 0.001), while this was not observed in healthy eyes (3.55 ± 0.54 vs 3.50 ± 0.45, respectively; p = 0.065).

It seems that, the use of ultrasound could be helpful in decreasing the number of unclear PLR through accurate detection and measurement of pupil size in injured eyes. Emergency physicians can consider ultrasonography as an accessible tool, complementary to physical examination for more accurate decision making in treatment of trauma patients. 
